# Usefulness of a novel sphincterotome for transpancreatic biliary sphincterotomy to achieve selective biliary cannulation in patients with Roux-en-Y gastrectomy

**DOI:** 10.1055/a-2063-3521

**Published:** 2023-04-21

**Authors:** Yuki Tanisaka, Masafumi Mizuide, Akashi Fujita, Rie Shiomi, Takahiro Shin, Kei Sugimoto, Shomei Ryozawa

**Affiliations:** Department of Gastroenterology, Saitama Medical University International Medical Center, Saitama, Japan

Selective biliary cannulation in patients with Roux-en-Y gastrectomy is considered technically difficult 1 2 . In cases where a guidewire is inserted into the pancreatic duct, transpancreatic biliary sphincterotomy is considered an effective method of achieving biliary cannulation 3 . Herein, we report a case of successful selective biliary cannulation via transpancreatic biliary sphincterotomy using a novel sphincterotome in a patient with Roux-en-Y gastrectomy.

A 78-year-old man with choledocholithiasis was referred to our facility. The patient previously underwent a Roux-en-Y gastrectomy. Computed tomography revealed stones in the common bile duct. Therefore, endoscopic retrograde cholangiopancreatography was performed using a short-type single-balloon enteroscope (SIF-H290; Olympus Marketing, Tokyo, Japan), with a working length of 152 cm and a working channel diameter of 3.2 mm 4 ( Video 1 ). After reaching the papilla, selective biliary cannulation was attempted. However, only pancreatic duct cannulation was achieved ( Fig. 1 ). As the pancreatic guidewire-assisted biliary cannulation was unsuccessful, transpancreatic biliary sphincterotomy was performed using a novel sphincterotome (CleverCut3V; KD-V410V-0720; Olympus Marketing). This device is dedicated to patients with surgically altered anatomy, such as in cases of Roux-en-Y gastrectomy ( Fig. 2 ) 5 . As the blade was easily adjusted from the 5 to the 6 o’clock position, an ideal incision for transpancreatic biliary sphincterotomy was achieved ( Fig. 3 ). The bile duct orifice was clearly observed at the incision site and selective biliary cannulation was easily achieved ( Fig. 4 ). Subsequently, endoscopic sphincterotomy was performed using the sphincterotome, followed by complete stone extraction ( Fig. 5 ).

**Video 1** Successful selective biliary cannulation via transpancreatic biliary sphincterotomy using a novel sphincterotome in a patient with Roux-en-Y gastrectomy.


**Fig. 1:**
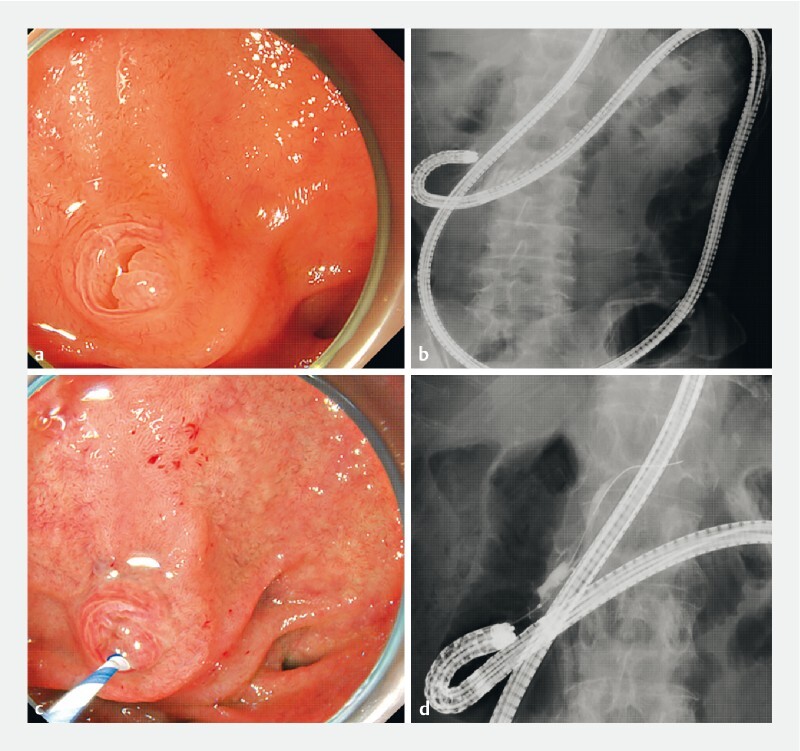
Endoscopic ( **a, c** ) and fluoroscopic ( **b, d** ) findings. **a, b** The single-balloon enteroscope reached the papilla. **c, d** Pancreatic duct cannulation was achieved.

**Fig. 2:**
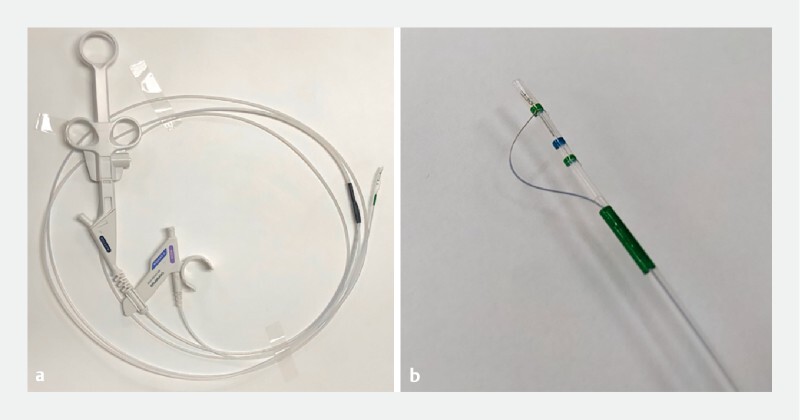
The novel sphincterotome (CleverCut3V; KD-V410V-0720; Olympus Marketing, Tokyo, Japan), with a working length of 240 cm for balloon enteroscopy-assisted endoscopic retrograde cholangiopancreatography in patients with surgically altered anatomy.

**Fig. 3:**
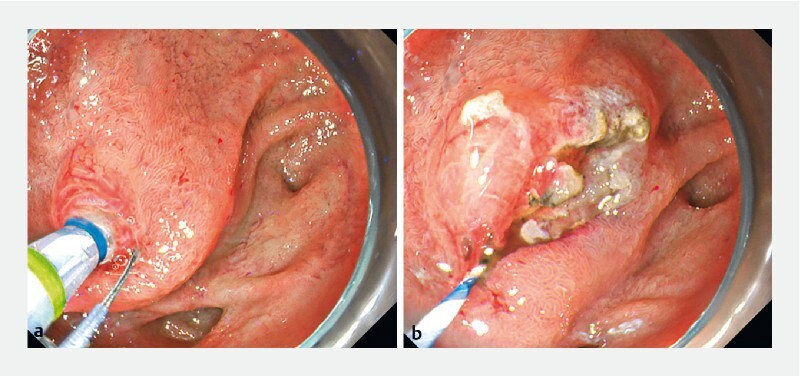
Endoscopic findings. As the blade was easily adjusted from the 5 to the 6 o’clock position, an ideal incision for transpancreatic biliary sphincterotomy could be achieved.

**Fig. 4:**
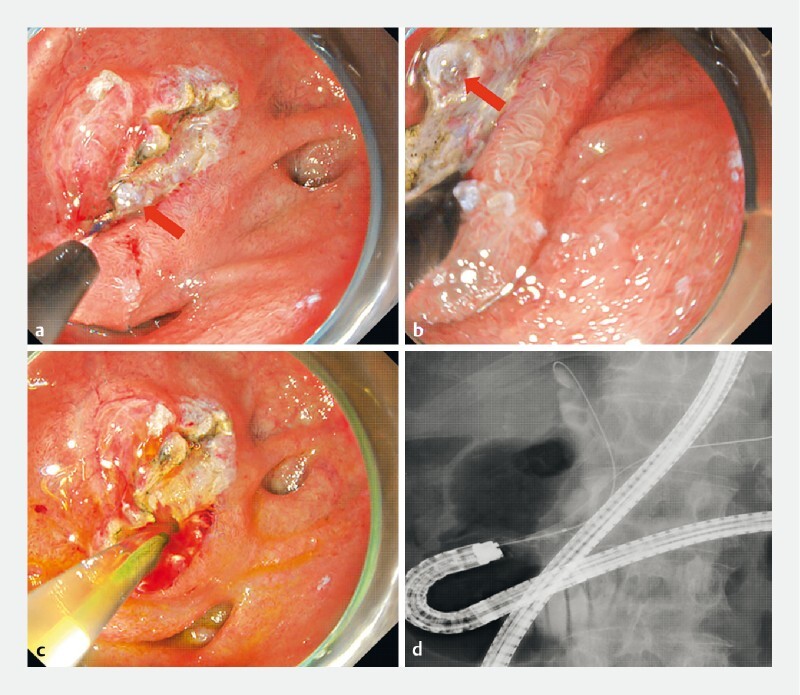
Endoscopic ( **a–c** ) and fluoroscopic ( **d** ) findings. The bile duct orifice (red arrow) was clearly observed at the incision site and selective biliary cannulation was easily achieved.

**Fig. 5:**
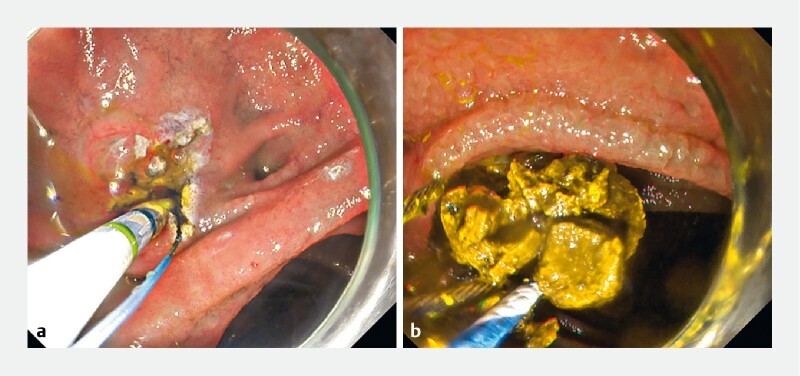
Endoscopic findings. Endoscopic sphincterotomy was performed using the sphincterotome, followed by complete stone extraction.

This novel sphincterotome could overcome the difficulty of adjusting the incision direction for sphincterotomy in patients with surgically altered anatomy. Therefore, it facilitates endoscopic sphincterotomy as well as transpancreatic biliary sphincterotomy in difficult biliary cannulation cases. This device could aid in the development of a safe and effective advanced selective biliary cannulation technique.

Endoscopy_UCTN_Code_TTT_1AR_2AC

## Notes

Endoscopy E-Videos

https://eref.thieme.de/e-videos
*E-Videos* is an open access online section of the journal *Endoscopy* , reporting on interesting cases and new techniques in gastroenterological endoscopy. All papers include a high-quality video and are published with a Creative Commons CC-BY license. Endoscopy E-Videos qualify for HINARI discounts and waivers and eligibility is automatically checked during the submission process. We grant 100% waivers to articles whose corresponding authors are based in Group A countries and 50% waivers to those who are based in Group B countries as classified by Research4Life (see: https://www.research4life.org/access/eligibility/ ). This section has its own submission website at https://mc.manuscriptcentral.com/e-videos
